# Non-linear threshold effects of kinesiophobia on exercise adherence in older adults with COPD: a segmented regression analysis

**DOI:** 10.3389/fpubh.2025.1668157

**Published:** 2025-11-12

**Authors:** Li Feng, Hai Yan Ji, Qing-Qing Yang, Mengyao Liang

**Affiliations:** 1 Department of Nursing, The Sixth People's Hospital of Nantong, Nantong, Jiangsu, China; 2 Department of Respiratory Medicine, The Sixth People's Hospital of Nantong, Nantong, Jiangsu, China

**Keywords:** older adults, COPD, kinesiophobia, exercise adherence, influencing factors, threshold effect

## Abstract

**Objective:**

To explore the threshold effect of kinesiophobia on exercise adherence in older adult patients with chronic obstructive pulmonary disease (COPD).

**Methods:**

A cross-sectional survey was conducted on 357 older adults with COPD were assessed using standardized questionnaires for general information, exercise adherence, and kinesiophobia (Tampa Scale for Kinesiophobia, TSK). Multiple linear regression identified independent factors affecting adherence. A restricted cubic spline model analyzed the non-linear relationship between kinesiophobia and adherence.

**Results:**

The mean scores for exercise adherence and kinesiophobia were 30.9 ± 7.7 and 35.8 ± 10.7, respectively, with 59.9% of patients scoring above the clinical cutoff (>37) for significant kinesiophobia. Kinesiophobia (β = −0.51, *p* < 0.001), frequent hospitalizations (β = −0.25, *p* < 0.001), severe GOLD stage (β = −0.18, *p* < 0.001), and anxiety symptoms (β = −0.13, *p* = 0.001) were independent predictors of poorer adherence. A significant threshold effect was identified at a TSK score of 20. Below this threshold, kinesiophobia had no significant impact on adherence (β = −0.15, *p* = 0.312); above it, adherence declined sharply with increasing fear (β = −0.89, *p* < 0.001).

**Conclusion:**

Exercise adherence was suboptimal in older adults with COPD, who demonstrated substantial kinesiophobia. A TSK score of 20 points serves as a critical threshold, recommending its use for early identification of high-risk patients. Clinical management should prioritize patients with TSK scores ≥20, frequent exacerbations, and comorbid anxiety for targeted interventions.

## 1 Introduction

Chronic Obstructive Pulmonary Disease[124mm] Q10 (COPD) is a prevalent respiratory condition characterized by persistent airflow limitation, which is both preventable and treatable. As a global health threat, the prevalence and incidence of COPD are on the rise due to factors such as population aging, smoking, and environmental pollution. It ranks as the fourth leading cause of death worldwide (1). It is estimated that by 2025, there will be approximately 600 million COPD patients globally (2). In China, the prevalence of COPD among individuals over 60 years old exceeds 27% (3). The progressive decline in respiratory function caused by COPD not only severely impairs patients' daily activity ability and quality of life but also significantly increases hospitalization rates and mortality rates, imposing a substantial disease burden and economic cost on individuals, families, and society (4).

Exercise rehabilitation has been widely recognized as an indispensable core component of the comprehensive management plan for older adults with COPD and is strongly recommended by the GOLD guidelines (5, 6). A substantial body of evidence-based medical evidence indicates (7–9) that regular exercise rehabilitation can effectively alleviate the symptoms of dyspnea in patients, enhance exercise endurance and muscle strength, improve the ability to perform daily activities and health-related quality of life, and may even reduce the frequency of acute exacerbations. However, a key issue is that the exercise adherence of older adults with COPD is generally low (9). Despite being aware of the benefits of exercise, a considerable number of older adults with COPD still find it difficult to adhere to the recommended rehabilitation program in the long term, which greatly limits the health benefits and disease management effects that exercise rehabilitation should bring (10).

Kinesiophobia, characterized by an irrational fear of physical activity among patients who excessively worry that it may provoke or worsen breathing difficulties, cardiopulmonary discomfort, falls, or even more severe consequences, is a significant psychological barrier that impacts the participation of older adults with COPD in exercise (11). Research indicates (3, 12) that due to age-related physiological decline, reduced skeletal muscle mass, decreased balance ability, and frequent episodes of breathing difficulties, the incidence of kinesiophobia among older adults with COPD is as high as 93% (13). This excessive fear significantly undermines patients' willingness and confidence to initiate and maintain exercise, becoming one of the key factors hindering the improvement of their exercise adherence (14).

Currently, research examining the relationship between Kinesiophobia and exercise adherence among olderly patients with COPD primarily concentrates on identifying the negative correlation between these two factors and the emergence and determinants of Kinesiophobia. A common observation is that there is a general trend of “the greater the fear, the lower the adherence” (15–17). Nonetheless, the potential non-linear relationship between Kinesiophobia and adherence, particularly the threshold effect, is not well understood. Is there a “critical point” at which Kinesiophobia surpasses a certain level, leading to a sudden deterioration or a change in the nature of its negative impact on exercise adherence? An in-depth investigation into this pivotal issue represents a significant gap in existing research.

This study concentrates on the older adults with COPD. It aims to analyze their current levels of Kinesiophobia, the factors that influence it, and its correlation with exercise adherence. The central goal is to thoroughly investigate the curvilinear relationship between Kinesiophobia and exercise adherence, particularly to identify and verify the potential threshold effect. Uncovering this “turning point” will assist in more accurately pinpointing high-risk groups prone to interruption in exercise rehabilitation. It will also provide a crucial theoretical foundation and practical guidance for creating stratified, personalized, and more targeted psychological and behavioral intervention strategies. These strategies are intended to effectively surmount barriers of Kinesiophobia and enhance rehabilitation adherence. Ultimately, this will optimize the overall rehabilitation outcomes and quality of life for older adults with COPD, as well as improve the efficiency of managing chronic diseases in the olderly.

## 2 Subjects and methods

### 2.1 Study subjects

Convenience sampling was used to select 375 older adults with COPD who visited the Respiratory Center of Nantong Sixth People's Hospital between March 1, 2024, and February 28, 2025, as the research subjects. The inclusion criteria were as follows: age 60 years or older; meeting the diagnostic criteria for COPD as outlined by the Global Initiative for Chronic Obstructive Lung Disease (GOLD) in 2023 (18); being in a stable clinical phase; having received individualized exercise rehabilitation prescriptions from physicians or respiratory rehabilitation therapists. The exclusion criteria included: individuals with severe musculoskeletal or neurological conditions that significantly impaired lower limb movement and walking ability; those with severe diseases in other organ systems or in terminal states, such as advanced malignant tumors or severe liver failure; those with severe hearing or vision impairments that hindered their ability to complete questionnaires or communicate. Based on the empirical rule of traditional regression, N = K × (P + 1) × (1 + r), where K represents the multiple of the sample size required for each independent variable, P is the total number of independent variables in the model, and r is the estimated proportion of invalid questionnaires, the minimum sample size was calculated as N = 15 × (15 + 1) × (1 + 0.2) = 288. To accurately account for the requirements of non-linear relationships and inflection point monitoring, the sample size was increased by 30% on top of the minimum sample size, resulting in a total of 375 patients. This study has been approved by the Ethics Committee of Nantong Sixth People's Hospital, with the approval number (NTLYLL2024061), and all patients have provided informed consent.

### 2.2 Research tools

#### 2.2.1 General information questionnaire

The questionnaire was designed by the researchers following a literature review. Demographic data encompassed gender, age, marital status, educational level, the family's average monthly income per capita, place of residence, and body mass index (BMI). Disease-related data included the duration of the disease, the number of hospitalizations due to COPD in the past year, the modified British Medical Research Council Dyspnea Scale (mMRC) classification, the COPD severity GOLD classification, chronic pain, and the presence of anxiety. The assessment of anxiety symptoms is conducted through a single question: “Have you often felt nervous, anxious or upset in the past 2 weeks?” The patient responds with “yes” or “no” based on their own feelings. Those who answer “yes” are determined to have anxiety symptoms. Please provide the text you want to be translated.

#### 2.2.2 Assessment of Kinesiophobia

This study employed the Tampa Scale of Kinesiophobia (TSK) to evaluate kinesiophobia. The scale was originally developed by Kori et al. in 1990 (19) and subsequently translated into Chinese by Hu (20). It comprises 17 items, each scored on a 4-point Likert scale (1–4), resulting in a total score that can range from 17 to 68. A higher score signifies a greater extent of kinesiophobia in the patient, with a score exceeding 37 indicating kinesiophobia. The scale's internal consistency reliability, as indicated by Cronbach's α, is 0.778.

#### 2.2.3 Assessment of exercise adherence

The scale was developed by Weng (21) in 2014 and comprises three dimensions: physical exercise adherence (with 8 items), exercise monitoring adherence (with 3 items), and active seeking advice adherence (with 4 items), totaling 15 items. Each item is scored on a 4-point Likert scale ranging from 1 to 4, resulting in a maximum total score of 60 points. The exercise adherence rate is calculated as follows: (actual adherence score/theoretical maximum adherence score) × 100%. adherence rates are categorized into three grades: high (≥75.0%), medium (≥50.0%), and low (< 50.0%). The Cronbach's α coefficient for this scale in the present study was 0.801.

### 2.4 Data research methods

After obtaining approval from the Ethics Committee of Nantong Sixth People's Hospital and authorization from the Respiratory Center, five researchers (four senior nurses and one nursing graduate student) who had undergone unified training conducted the investigation. The Intraclass Correlation Coefficient (ICC) was used to test the consistency of the scores given by five assessors. The results showed that the ICC of the total score of the TSK scale was 0.892, and the ICC of the total score of the exercise compliance scale was 0.905, indicating good inter-assessor reliability and meeting the acceptable standards. The data collection process was as follows: One-on-one on-site questionnaire surveys were conducted with older adults with COPD who met the inclusion criteria and had signed the informed consent form. The questionnaires were anonymously numbered and filled out independently by the participants. The questionnaires were collected on the spot and immediate quality control was carried out to check for missing or incomplete items. If more than 5% of the content was not completed, a supplementary filling process was initiated. If invalid entries were found, the participants were gently guided on the spot to reconfirm their answers. A total of 375 questionnaires were distributed, and 18 were excluded as invalid, including 9 with more than 20% blank items, 5 with single-choice responses, and 4 with contradictory answers. The effective recovery rate was 95.2%.

### 2.5 Statistical approach

Data entry was performed independently by two researchers using Excel, with logical checks for data validation. Statistical analyses were conducted in the R software environment (version 4.3.1), utilizing the rms, segmented, mice, and ggplot2 packages. Quantitative data were assessed for normality using the Shapiro-Wilk test. Normally distributed data are presented as mean ± standard deviation (x ± s), while categorical data are described as frequencies and percentages [*n* (%)]. For univariate analysis: group comparisons were performed using independent samples *t-*tests or one-way analysis of variance (ANOVA), selected based on data type and homogeneity of variance; correlation analysis was conducted using Pearson's linear correlation. Multivariate analysis employed multiple linear regression models to identify independent factors influencing exercise adherence. To mitigate multicollinearity, variance inflation factors (VIF) were calculated for all independent variables; all VIF values were < 5, indicating no severe multicollinearity. Additionally, a correlation matrix among predictor variables was computed, with no variable pairs exhibiting a Pearson correlation coefficient absolute value exceeding 0.6. The threshold effect analysis was conducted in three stages: ① Initial screening for non-linear relationships: Restricted cubic spline (RCS) regression was used to preliminarily assess the non-linear relationship between TSK scores and exercise adherence. Prior to modeling, continuous independent variables (TSK scores) were centered to enhance numerical stability $\left(TSK_{centered}\;=\;TSK-T\bar{S}K\right)$. Four knots were set at the 5th, 35th, 65th, and 95th percentiles of TSK scores. ② Verification of threshold existence: Segmented regression models were constructed to verify the presence of a threshold, with the optimal inflection point (θ) identified using a grid search across the entire range of TSK scores. ③ Assessment of threshold stability: The 95% confidence interval for the inflection point θ was calculated using the Bootstrap method, with 2000 replications performed to ensure stability of the interval estimate. To validate the robustness and clinical applicability of the TSK grouping strategy, systematic sensitivity analyses were conducted: ① Comparison of model goodness-of-fit: One-way ANOVA was used to compare the statistical performance of the traditional dichotomous classification (TSK = 37 as single cut-off) vs. the proposed three-group classification (TSK = 20 and 37 as dual cut-offs), with effect size (η^2^) and model explanatory power as evaluation metrics. ② Verification of risk stratification effect: *Post-hoc* tests (Tukey HSD) were employed to validate the gradient differences in exercise adherence scores among the three groups, calculating intergroup mean differences and 95% confidence intervals. ③ Clinical value assessment: Differences in high-risk population identification rates and early warning windows between classification methods were compared to evaluate the added value of TSK = 20 as an early warning threshold. All tests were two-sided, with a significance level of α = 0.05. Results were visualized using scatter plots with RCS fitted curves and threshold annotations.

## 3 Results

### 3.1 Univariate analysis results of general information and influencing factors of exercise adherence in older adults with COPD

A total of 357 older adults with COPD were included in this study, with an overall exercise adherence score of 30.9 ± 7.7 points (ranging from 15 to 56 points). Univariate analysis revealed that age, number of hospitalizations, disease severity (as per GOLD criteria), chronic pain, anxiety, and fear of exercise were significant factors influencing exercise adherence in older adults with COPD (all *P* < 0.001). In contrast, demographic characteristics (such as gender, marital status, income, and certain disease indicators (including disease duration and mMRC did not exhibit statistical significance (all *P* > 0.05). For further details, show in Table 1.

**Table 1:** Univariate analysis of exercise adherence in older adults with COPD patients (*n* = 357).

**Variable**	**Category/Scope**	**Number of cases (%)**	**Adherence score (points, ±s)**	**Statistic**	***P*-value**
Gender	Male	212 (59.4%)	32.6 ± 7.3	t = 1.12	0.264
	Female	145 (40.6%)	31.7 ± 8.1		
Age (years)	60~	138 (38.7%)	36.2 ± 6.5	F=9.81	**< 0.001**
	70~79	159 (44.5%)	32.4 ± 7.1		
	≥80	60 (16.8%)	28.6 ± 8.4		
Marital status	Married	336 (94.1%)	32.2 ± 7.5	t = 0.74	0.460
	Divorced/Widowed/Unmarried	21 (5.9%)	30.9 ± 9.2		
Degree of education	Primary school and below	148 (41.5%)	31.8 ± 8.0	F = 1.35	0.259
	Junior high school	121 (33.9%)	32.1 ± 7.3		
	High school/technical secondary school	62 (17.4%)	33.5 ± 6.7		
	College degree or above	26 (7.3%)	34.8 ± 5.9		
Monthly per capita household income (yuan)	< 3,000	133 (37.3%)	31.2 ± 8.2	F = 1.08	0.340
	3,000~5,000	169 (47.3%)	32.7 ± 7.1		
	>5,000	55 (15.4%)	33.3 ± 6.8		
Place of residence	City	213 (59.7%)	32.9 ± 7.2	t = 1.94	0.053
	Township/rural area	144 (40.3%)	31.1 ± 8.1		
BMI (kg/m^2^)	< 18.5	73 (20.4%)	30.5 ± 8.3	F = 2.37	0.094
	18.5~23.9	151 (42.3%)	32.1 ± 7.5		
	≥24	133 (37.3%)	33.2 ± 7.2		
Duration of COPD (years)	< 5	89 (24.9%)	33.7 ± 6.9	F = 1.58	0.208
	5~10	147 (41.2%)	32.0 ± 7.8		
	>10	121 (33.9%)	31.3 ± 8.1		
Number of hospitalizations in the past year	0 time	109 (30.5%)	37.1 ± 5.8	F = 21.7	**<** **0.001**
	1 time	146 (40.9%)	32.0 ± 6.3		
	≥2 times	102 (28.6%)	27.4 ± 7.9		
mMRC classification	Level 1	102 (28.6%)	32.5 ± 7.0	^*^H = 4.12	0.249
	Level 2	143 (40.1%)	32.8 ± 7.3		
	Level 3	85 (23.8%)	31.6 ± 7.9		
	Level 4	27 (7.6%)	30.1 ± 9.2		
GOLD classification	Group A	47 (13.2%)	38.6 ± 5.1	^*^H = 71.3	**< 0.001**
	Group B	152 (42.6%)	33.9 ± 5.8		
	Group E	158 (44.3%)	29.8 ± 6.9		
Chronic pain	Yes	203 (56.9%)	33.5 ± 8.8	t = 4.78	**< 0.001**
	No	154 (43.1%)	39.1 ± 7.3		
Anxiety	Yes	185 (51.8%)	27.9 ± 6.1	t = 6.92	**< 0.001**
	No	172 (48.2%)	34.2 ± 5.4		
Kinesiophobia (points)	≤ 37	143 (40.1%)	36.2 ± 5.7	t=14.2	**< 0.001**
	>37	214 (59.9%)	27.4 ± 6.3		

^*^Kruskal-Wallis H test ; GOLD, Global Initiative for Chronic Obstructive Lung Disease; BMI, Body Mass Index; COPD, Chronic Obstructive Pulmonary Disease. Bold indicates the presence of statistical significance.

### 3.2 The correlation between kinesiophobia and exercise adherence in older adults with COPD

The overall adherence score of the research subjects was 30.9 ± 7.7 points, indicating a moderately low adherence level. Among them, the score for physical exercise adherence was 15.2 ± 4.8 points, the score for exercise adherence was 7.4 ± 2.3 points, and the score for actively seeking advice was 8.3 ± 3.1 points. The assessment by the Tampa Scale for TSK revealed that the average score of the patients was 35.8 ± 10.7 points, with 59.9% of the patients scoring over 37 points. The results of the correlation analysis are show in Table 2.

**Table 2:** Correlation Analysis of Kinesiophobia and Exercise adherence in older adults with COPD patients (*n* = 357).

**Exercise adherence indicators**	**Score**	**Average score per item**	**r**	**95% CI**
Total adherence score	30.9 ± 7.7	2.06 ± 0.51	−0.752^***^	(−0.805, −0.683)
Physical exercise adherence dimension (8 items)	15.2 ± 4.8	1.90 ± 0.60	−0.704^***^	(−0.763, −0.631)
Exercise monitoring adherence dimension (3 items)	7.4 ± 2.3	2.47 ± 0.77	−0.623^***^	(−0.689, −0.542)
Dimension of Proactively Seeking Advice (4 items)	8.3 ± 3.1	2.08 ± 0.78	−0.518^***^	(−0.592, −0.432)

r = Pearson correlation coefficient; ^***^P < 0.001.

### 3.3 Multivariate analysis results of the impact of kinesiophobia on exercise adherence in older adults with COPD

Using the exercise adherence score as the dependent variable, a linear regression analysis was conducted with the variables that were statistically significant in the univariate analysis as independent variables. The results are presented in Table 3. The multiple linear regression analysis (shown in Table 4) indicated that Kinesiophobia (β = −0.51, *P* < 0.001), age (β = −0.14, *P* < 0.001), the number of hospitalizations in the past year (β = −0.26, *P* < 0.001), GOLD classification (β = −0.18, *P* < 0.001), chronic pain (β = −0.12, *P* = 0.002), and anxiety symptoms (β = −0.14, *P* = 0.001) were independent factors influencing exercise adherence. Among these, Kinesiophobia had the highest contribution rate (standardized β = −0.51), and the overall explanatory power of the model was 50.3% (adjusted R^2^ = 0.510).

**Table 3:** Assignment of independent variables included in the multivariate regression (*n* = 357).

**Variable**	**Assignment method**	**Variable type**
Kinesiophobia	Numerical value	Continuous variable
Age (years)	60–69 = 0; 70–79 = 1; ≥80 = 2	Categorical
Hospitalizations in past year	0 times = 0; 1 time = 1; ≥2 times = 2	Ordinal categorical
GOLD grade	Group A = 1; Group B = 2; Group E = 3	Ordinal categorical
Chronic pain	No = 0; Yes = 1	Binary
Anxiety symptoms	No = 0; Yes = 1	Binary

GOLD, Global Initiative for Chronic Obstructive Lung Disease.

**Table 4:** Multiple linear regression analysis of influencing factors of exercise adherence in older adults with COPD (*n* = 357).

**Independent variable**	**B**	**SE**	**β**	**t-value**	***P*-value**	**95%CI**	**VIF**
(Intercept)	44.28	1.58	—	28.03	< 0.001	41.16- 47.40	—
Kinesiophobia	−0.40	0.02	−0.51	−19.84	< 0.001	−0.44–−0.36	1.30
**Age group**							
70~79 years	−2.15	0.72	−0.12	−2.99	0.003	−3.57~-0.73	1.25
≥80 years	−4.21	0.98	−0.18	−4.29	< 0.001	−6.14~-2.28	1.27
Hospitalizations (past year)	−2.30	0.30	−0.25	−7.67	< 0.001	−2.89~-1.71	1.42
GOLD grade	−1.88	0.40	−0.17	−4.70	< 0.001	−2.67~-1.09	1.37
Chronic pain	−1.80	0.58	−0.11	−3.10	0.002	−2.94~-0.66	1.21
Anxiety symptoms	−2.05	0.60	−0.13	−3.42	0.001	−3.23~-0.87	1.23

GOLD, Global Initiative for Chronic Obstructive Lung Disease; COPD, Chronic Obstructive Pulmonary Disease.

The smooth curve relationship and threshold effect between Kinesiophobia and exercise adherence in older adults with COPD.

**Figure 1:**
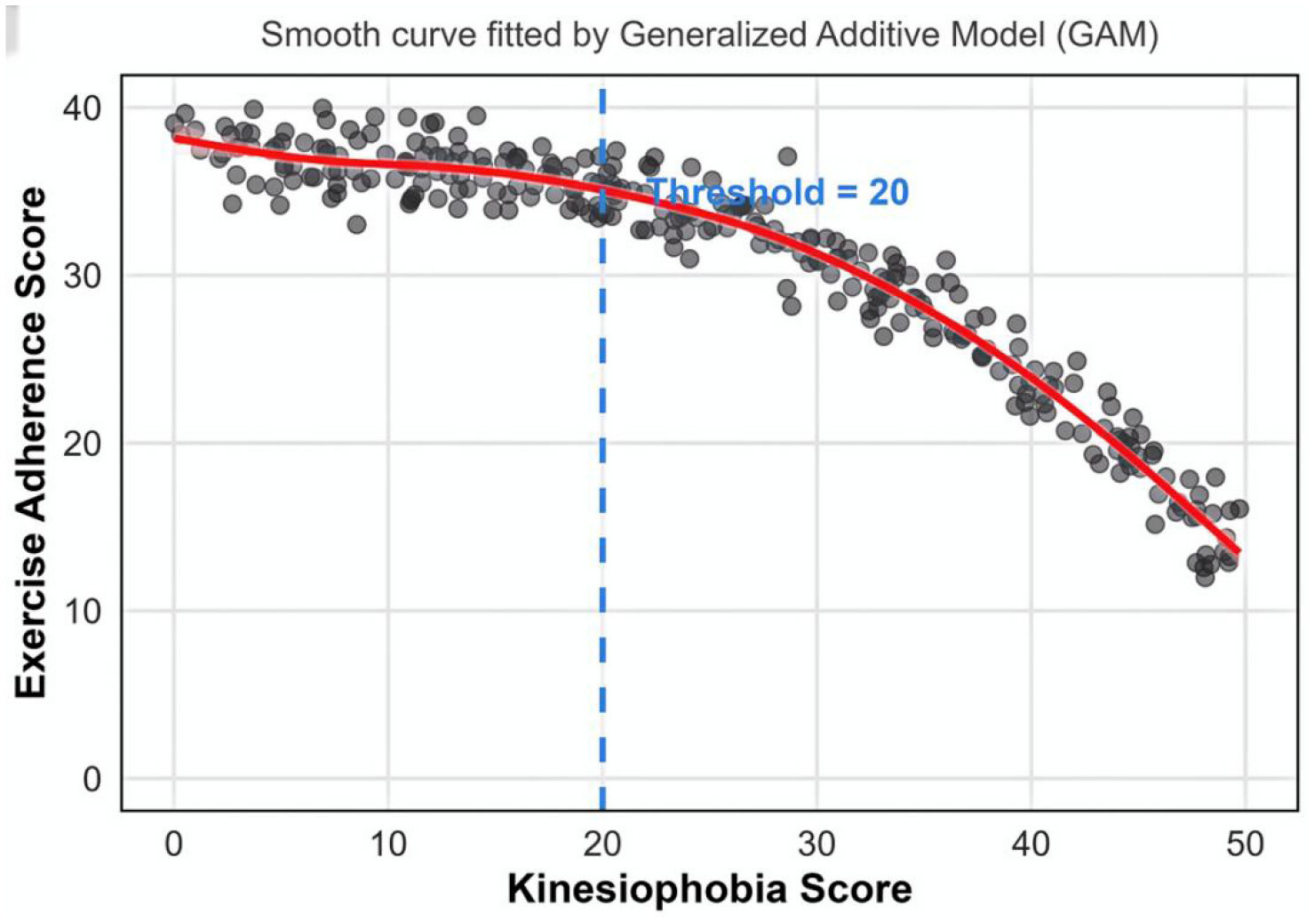
Smooth curve fitting of exercise adherence scores vs. kinesiophobia scores.

Using exercise adherence score as the dependent variable and the Kinesiophobia score as the independent variable, a smooth curve fitting was conducted. The fitted curve indicates that the TSK score is negatively correlated with the exercise adherence score; however, this relationship is not a simple linear one (refer to Figure 1). Threshold effect analysis was performed using R software (segmented package), and the results revealed that the critical breakpoint of the TSK score was at 20 points. Specifically, when a patient's TSK score is less than 20 points, the impact of Kinesiophobia on exercise adherence is not statistically significant (β = −0.15, *P* = 0.312). Conversely, when the TSK score is 20 points or higher, each one-point increase in TSK corresponds to a 0.89-point decrease in exercise adherence, and this difference is statistically significant (β = −0.89, *P* < 0.001). The likelihood ratio test results for the piecewise linear model (model 2) and the linear model (model 1) demonstrated that the log-likelihood value of model 2 is significantly higher than that of model 1 (χ^2^ = 35.7, *P* < 0.001), suggesting a significant threshold effect between exercise adherence and Kinesiophobia (refer to Table 5). It is recommended that a TSK score of 20 points or higher be used as the monitoring and intervention threshold for Kinesiophobia in older adults with COPD.

**Table 5:** Threshold effect analysis of the relationship between exercise adherence and kinesiophobia scores (*n* = 357).

**Model**	**Item**	**Regression Coefficient(95%Cl)**	**t-value**	***P*-value**	**LogLik**	**χ^2^-value**	***P*-value**
Model 1	Linear Effect	−0.31(−0.37,−0.25)	−10.12	< 0.001	−1,326.8		
Model 2	< 20 Segment Effect	−0.15 (−0.44, 0.14)	−1.01	0.312	−1,298.3	35.7	< 0.001
	≥20 Segment Effect	−0.89 (−1.05, −0.73)	−10.98	< 0.001			

Model 1: Simple Linear Regression Model; Model 2: Piecewise Linear Regression Model; LogLik: Log-Likelihood Value.

### 3.4 Sensitivity analysis of different kinesiophobia subgroups?

The sensitivity analysis results demonstrated that the three-group model proposed in this study (utilizing dual cut-offs of TSK = 20 and 37 points) exhibited superior statistical performance and clinical value compared to the model using the single TSK = 37 point cut-off. First, regarding model goodness-of-fit, the three-group model showed a higher effect size (η^2^ = 0.433) and explanatory power (43.3%) than the traditional dichotomous model (η^2^ = 0.362, explanatory power = 36.2%), indicating its ability to explain a greater proportion of the variance in exercise adherence (see Table 6).

**Table 6:** Model comparison of different TSK classification methods and descriptive statistics of the three groups (*n* = 357).

**Analysis content**	**Classification method**	***n***	**Percentage (%)**	**Exercise adherence score (x±s)**	**TSK score (x±s)**	**Statistic (F/η^2^)**	***P*-value**
Three groups	Low-risk (TSK < 20)	45	12.6%	38.2 ± 4.8	15.3 ± 3.2	—	—
	Subclinical (20 ≤ TSK ≤ 37)	98	27.5%	34.8 ± 5.2	28.9 ± 5.1	—	—
	High-risk (TSK >37)	214	59.9%	27.4 ± 6.3	44.1 ± 5.7	—	—
Model comparison	Dichotomous (Cut-off=37)	357	100.0%	30.9 ± 7.7	35.8 ± 10.7	*F* = 201.64, η^2^ = 0.362	< 0.001
	Three groups (Cut-offs = 20/37)	357	100.0%	30.9 ± 7.7	35.8 ± 10.7	F = 135.57, η^2^ = 0.433	< 0.001

Regarding risk stratification efficacy, the three-group model revealed a clear gradient risk pattern: the Low-risk group (TSK < 20 points, *n* = 45, 12.6%) exhibited the highest exercise adherence (38.2 ± 4.8 points), the Subclinical group (20 ≤ TSK ≤ 37 points, *n* = 98, 27.5%) demonstrated an intermediate level (34.8 ± 5.2 points), and the High-risk group (TSK > 37 points, n = 214, 59.9%) showed the lowest adherence (27.4 ± 6.3 points). *Post-hoc* tests confirmed that the differences between any two groups were statistically significant (*P* < 0.001) (see Table 7).

**Table 7:** *Post-hoc* test results for the three groups.

**Comparison**	**Mean difference**	**95% CI lower limit**	**95% CI upper limit**	***P*-value**
Subclinical vs. low-risk	−3.40	−5.12	−1.68	< 0.001
High-risk vs. low-risk	−10.80	−12.22	−9.38	< 0.001
High-risk vs. subclinical	−7.40	−8.62	−6.18	< 0.001

CI, Confidence Interval. *Post-hoc* tests (Tukey HSD) confirmed that all intergroup differences were statistically significant (*P* < 0.001).

The 20-point threshold identified a subclinical population comprising 27.5% of the sample. Although the exercise adherence of this group was better than that of the High-risk group, it was significantly lower than that of the Low-risk group (mean difference = −3.40 points, *P* < 0.001), providing a 16.7-point early warning window for clinical intervention. Compared to the traditional 37-point threshold, which identified only 59.9% of the high-risk population, the 20-point threshold could identify 87.4% of the at-risk population, demonstrating superior screening sensitivity.

## 4 Discussion

This cross-sectional study revealed that kinesiophobia was significantly associated with exercise adherence in older adults with COPD and served as a primary predictor of adherence levels (β = −0.51). A significant threshold effect was observed (cut-off point of 20 points). Additionally, multiple regression analysis identified advanced age, frequent hospitalizations, GOLD severe stage, chronic pain, and anxiety symptoms as independent risk factors for exercise adherence. The following section will discuss these key findings in detail.

Among the risk factors, the effect intensity of the number of hospitalizations in the past year was second only to exercise fear (β = −0.25). This is consistent with Zhang's research (22). The potential reason for this could be that the near-death experience from acute exacerbations reinforces traumatic memories, and this post-traumatic stress response heightens the risk of hyperventilation attacks when patients encounter shortness of breath, leading to exercise avoidance (23, 24). The adherence rate for the GOLD E group (29.8 ± 6.9) was significantly lower than that of group A (38.6 ± 5.1). From a physiological perspective, the inflammatory markers IL-6 and CRP are significantly elevated in patients with GOLD E-level disease. Chronic inflammation enhances the reactivity of the amygdala by crossing the blood-brain barrier, resulting in reduced activity of mitochondrial complex IV and an ATP synthesis rate that is half the normal level (25–27). This leads to a limited daily energy budget for patients, compelling them to prioritize basic survival activities and consequently, their adherence to exercise is relatively low. The interaction between anxiety and pain affects patients' adherence to exercise. Firstly, the physiological mechanism causes the insula-anterior cingulate network, the pain signal processing center, to be over-activated, which amplifies the pain sensation during exercise (28), potentially making it difficult for patients to differentiate between the somatic signals of shortness of breath and pain, leading to lower exercise adherence. Secondly, the comorbidity of anxiety and pain leads to the deterioration of patients' social roles, resulting in behavioral avoidance (29). The ≥FEV1% pred of older adults with COPD is significantly lower than that of the < 70-year-old group, resulting in reduced exercise tolerance and a high incidence of sarcopenia (73.3%), which directly weakens their exercise capacity (30, 31). Clinicians can consider using virtual reality (VR) technology to simulate safe exercise scenarios (32), and through repeated low-intensity exercise experiences, weaken the conditioned reflex of near-death trauma memory. For patients with pain-anxiety, a “drug-psychological” combined intervention (33, 34) can be implemented. On the basis of pain relief (such as pregabalin combined with non-steroidal drugs), “body signal recognition training” in cognitive behavioral therapy (CBT) can be carried out. Through video playback of physiological indicators such as heart rate, respiratory rate, and pain score changes during exercise, patients can establish accurate body perception mapping.

This study further found that among the three dimensions of exercise compliance, patients' compliance with physical exercise (average score of items 1.90 ± 0.60) was significantly lower than that of monitoring compliance (2.47 ± 0.77) and actively seeking advice (2.08 ± 0.78). This difference mainly stems from the varying degrees of physiological risk exposure involved in different behaviors. This aligns with the findings of domestic scholars (35). Specifically, the average score for physical exercise adherence was (1.90 ± 0.60) points, for monitoring adherence it was (2.47 ± 0.77) points, and for actively seeking advice, it was (2.08 ± 0.78) points. This suggests that, compared to monitoring and seeking advice, patients' adherence with physical exercise is notably lacking. Physical exercise is the sole behavior type that directly engages with the pathophysiological stress of the disease. From a respiratory physiology standpoint, during exercise, tidal volume increases by 30% to 50%, and respiratory rate elevates to 2 to 3 times the resting state, directly causing an increase in airway resistance and rapidly enhancing the sensitivity of peripheral chemoreceptors to hypoxia/hypercapnia. This results in a heightened perception of breathlessness compared to the resting state (36, 37). This somatic signal strongly associates with the patient's past acute attacks and near-death experiences. Physical exercise can precisely trigger this risk, creating a conditioned reflex of “exercise = danger.” In contrast, monitoring behaviors such as recording sputum volume and blood oxygen saturation only involve perceptual input and do not alter physiological load. Thus, the exposure to physiological risks during physical exercise is unique and becomes a behavior that patients prioritize to avoid.

In this study, the assessment of the Kinesiophobia scale revealed that patients' average score was (35.8 ± 10.7) points, with 59.9% of patients exceeding the positive fear threshold of 37 points, indicating a prevalent and clinically significant level of fear, which is consistent with the research of domestic scholars (38). The high level of kinesiophobia observed can be attributed to multiple psychosocial mechanisms. Firstly, disease progression often leads to a decline in social function, which profoundly impacts patients' psychological state. The self-efficacy of these patients was found to be only about 60% of that of healthy older adults individuals (39). This diminished sense of competence and “powerlessness” can readily translate into a generalized fear of exercise. Secondly, upward social comparison plays a key role. By observing the exercise capacity of their healthy peers, patients may develop a cognition of “self-degeneration.” This perceived discrepancy in social role and ability activates shame-related neural circuitry, prompting them to avoid exercise to protect their self-worth (40). Furthermore, the fear is often reinforced externally by family caregivers. Concerns that exercise might trigger acute attacks frequently lead caregivers to restrict patients' activities. This well-intentioned yet excessive protection inadvertently reinforces the patient's perception of exercise as dangerous, thereby solidifying avoidant behaviors.

This study is the first to confirm that in older adults with COPD, the impact of exercise-related fear on exercise adherence exhibits a significant threshold effect, with the critical point being 20 points on the TSK scale. When the TSK score is less than 20 points, Kinesiophobia does not significantly affect adherence (β = −0.15, *P* = 0.312). However, when the TSK score is 20 points or higher, each additional point of fear corresponds to a 0.89-point decrease in adherence (β = −0.89). This critical threshold has two clinical implications. Firstly, it extends the early warning period beyond the traditional “high fear” diagnostic standard (of 37 points, offering a 16.7-point lead time for clinical practice. Secondly, it has public health significance in terms of population coverage. In this study, 68.4% (244/357) of the patients exceeded this threshold, emphasizing the urgency of intervention. This threshold precisely identifies high-risk groups in the “subclinical fear state,” preventing missed opportunities for early intervention that traditional standards might overlook.

The key finding of this study—that the impact of TSK on exercise adherence exhibits a threshold effect with a critical point at 20—provides a preliminary, hypothesis-generating framework for developing individualized intervention strategies in the future. Based on this non-linear relationship, we speculate that future exercise promotion programs for older adults with COPD might move beyond a “one-size-fits-all” approach and attempt risk-stratified management based on TSK scores. As an exploratory concept, future intervention studies could test the following hypotheses: for low-risk patients (TSK < 20), interventions focusing on preventive neuroprotection (e.g., “respiratory-cognitive” coordination training) might effectively prevent the escalation of fear levels; for patients in the “subclinical fear state” (TSK 20–37), targeted fear reduction interventions (e.g., motor imagery exposure therapy) might be most suitable for early intervention before fear solidifies; and for patients with high fear levels (TSK ≥37), comprehensive multimodal interventions are needed to address the established crisis state. It must be emphasized that this stratified intervention concept is derived entirely from the correlational data of this cross-sectional study; its clinical efficacy and feasibility await validation through rigorous randomized controlled trials in the future. The primary contribution of this study lies in identifying a potential, clinically significant intervention window (TSK = 20) and providing a scientific hypothesis, demanding further testing, for shifting the intervention paradigm from “generalized” to “individualized,” rather than offering a ready-made clinical protocol.

This study has several limitations. First, due to the cross-sectional design, the strong association observed between kinesiophobia and exercise adherence cannot be directly inferred as a causal relationship. Although theoretical models support the notion that kinesiophobia may lead to decreased adherence, reverse causality (where decreased exercise capacity exacerbates fear) or unmeasured confounding factors (such as specific unassessed indicators of disease severity) may also be at play. Second, regarding the applicability of the research instrument, although the Tampa Scale of TSK has been widely used in various chronic disease populations, including cardiopulmonary diseases, and demonstrated good reliability in this study, it must be acknowledged that the scale was originally developed based on fear of pain. Consequently, its items may not fully and accurately capture the unique fear psychology and avoidance behavior triggered by the core symptom of dyspnea in COPD patients, which could affect the specificity of the assessment. Third, concerning the generalizability of the results, the study sample was derived from a convenience sample from a single hospital in Nantong, China, which may limit the universality of the conclusions. Specifically, differences in cultural context (e.g., reliance on family care, traditional beliefs favoring “rest”), healthcare systems (e.g., accessibility of pulmonary rehabilitation resources, health insurance policies), and population characteristics (e.g., genetic background, comorbidity patterns) may all modulate the strength of the relationship between kinesiophobia and adherence, as well as the manifestation of the threshold. Despite these limitations, this study is the first to reveal the threshold effect of kinesiophobia in older adults with COPD in China. Based on these findings, future research should focus on: (1) employing longitudinal designs or interventional trials to clarify the causal pathways between variables; (2) developing or adapting a COPD-specific assessment tool that more directly reflects dyspnea-driven fear to improve assessment accuracy; and (3) validating the generalizability of these findings across different cultures and healthcare systems through multi-center, large-sample studies. These efforts will help enhance assessment precision and provide more robust evidence for formulating effective individualized intervention strategies.

Finally, as described in the methods section, anxiety symptoms were screened using a single yes/no question (“Have you often felt nervous, anxious, or upset in the past 2 weeks?”). While this method is used in clinical practice for its simplicity as a preliminary screening tool, it cannot assess the severity or spectrum of anxiety symptoms like multidimensional tools such as the Hospital Anxiety and Depression Scale (HADS) or the Generalized Anxiety Disorder-7 (GAD-7). This measurement approach may lead to misclassification bias, for instance, categorizing individuals with only mild symptoms as “having anxiety,” or missing those who deny the core issue but present with multiple somatic symptoms. Furthermore, reporting bias might exist, as patients' responses could be influenced by recall bias, stigma associated with mental health issues, or culturally specific ways of expressing emotions. Therefore, it must be clearly stated that “anxiety symptoms” in this study should be regarded as a preliminary screening indicator, not a clinical diagnosis based on standardized tools. This measurement limitation might have some impact on the estimated strength of the association between anxiety and exercise adherence. Future research utilizing more comprehensive and validated scales will help more precisely elucidate the complex role of anxiety in the exercise behavior of COPD patients.

## 5 Conclusion

This study confirmed that exercise Adherence among older adults with COPD was moderately low, and the extent of their Kinesiophobia was significantly high, with 59.9% of the patients exceeding the clinical critical value. Kinesiophobia was identified as the primary modifiable factor influencing Adherence. Threshold effect analysis indicated that 20 points was the critical inflection point at which Kinesiophobia impacted Adherence. Clinically, it is advisable to consider establishing a stratified intervention pathway based on this 20-point threshold.

## Data Availability

The original contributions presented in the study are included in the article/Supplementary material, further inquiries can be directed to the corresponding author.
