# Correction: Non-linear threshold effects of kinesiophobia on exercise adherence in older adults with COPD: a segmented regression analysis

**DOI:** 10.3389/fpubh.2025.1746431

**Published:** 2025-12-04

**Authors:** Li Feng, Hai Yan Ji, Qing-Qing Yang, Mengyao Liang

**Affiliations:** 1 Department of Nursing, The Sixth People's Hospital of Nantong, Nantong, Jiangsu, China; 2 Department of Respiratory Medicine, The Sixth People's Hospital of Nantong, Nantong, Jiangsu, China

**Keywords:** older adults, COPD, kinesiophobia, exercise adherence, influencing factors, threshold effect

Author Mengyao Liang was erroneously spelled as Liang Yao.

The original version of this article has been updated.

