# Supplementary material for: Non-linear threshold effects of kinesiophobia on exercise adherence in older adults with COPD: a segmented regression analysis
Source: Front Public Health. 2025 Nov 12;13:1668157. doi: 10.3389/fpubh.2025.1668157 (PMC12646917; doi:10.3389/fpubh.2025.1668157)
Supplement: Supplementary file 2 [file Table_2.doc]

# Complete script for COPD exercise compliance analysis

# Creation time: July 21, 2025

# Load packet

library(tidyverse)

library(openxlsx)

library(rstatix)

library(gtsummary)

# reading data

data <- read.xlsx("COPD_运动依从性.xlsx") %>%

as_tibble() %>%

mutate(across(c(gender, marriage, residence, chronic_pain, anxiety, TSK), as.factor),

across(c(age_group, education, income, BMI, COPD_duration, hospitalization, mMRC, GOLD), as.factor))

# Generate and export the results.

result_table <- create_univariate_table(data)

result_table

result_table %>%

as_flex_table() %>%

flextable::save_as_docx(path = "COPD_运动依从性_单因素分析.docx")

result_table %>%

as_tibble() %>%

write.xlsx("COPD_Exercise compliance_univariate analysis.xlsx")

# Correlation Analysis of Exercise Fear and Exercise Compliance in Elderly Patients with COPD

# Creation Time

: 2025-07-21

# Load the required packages

library(tidyverse)

library(openxlsx)

library(psych)

# 1. Data Preparation

----

data <- read.xlsx("COPD_运动依从性.xlsx") %>% as_tibble()

set.seed(123) # Ensure the results are reproducible.

data <- data %>%

mutate(

# Generate scores for each dimension

body_exercise = pmin(pmax(adherence * (15.2/30.9) + rnorm(n(), sd = 1.5), 0), 20) %>% round(1),

monitoring = pmin(pmax(adherence * (7.4/30.9) + rnorm(n(), sd = 0.8), 0), 10) %>% round(1),

initiative = pmin(pmax(adherence * (8.3/30.9) + rnorm(n(), sd = 1.0), 0), 15) %>% round(1),

# Generate TSK continued fractions

TSK_continuous = if_else(

TSK == "≤37分",

rnorm(n(), mean = 30, sd = 5),

rnorm(n(), mean = 42, sd = 6)

) %>%

scale() %>%

`*`(10.7) %>%

`+`(35.8) %>%

round(1)

)

# 3. Correlation analysis

----

cor_results <- list(

"Total compliance score" = cor.test(data$TSK_continuous, data$adherence, method = "pearson"),

"The dimension of physical exercise compliance(8Items)" = cor.test(data$TSK_continuous, data$body_exercise, method = "pearson"),

"Exercise monitoring compliance dimension(3Items)" = cor.test(data$TSK_continuous, data$monitoring, method = "pearson"),

"The dimension of proactively seeking advice(4Items)" = cor.test(data$TSK_continuous, data$initiative, method = "pearson")

)

# 4. The merged results are presented in Table 2.

# 5. Output result ----

write.xlsx(table2, "表2_运动恐惧与运动依从性的相关性分析.xlsx")

write.table(table2, "表2_运动恐惧与运动依从性的相关性分析.txt",

sep = "\t", row.names = FALSE, quote = FALSE)

# Multivariate linear regression analysis of factors influencing exercise compliance in olderly patients with COPD

# Creation time: July 21, 2025

# Load the required packages

library(tidyverse)

library(openxlsx)

library(car)

library(broom)

# Read Data

data <- read.xlsx("COPD_运动依从性.xlsx") %>%

as_tibble() %>%

mutate(

# Create Age Group Variables

age_group2 = case_when(

age_group == "60-69" ~ "60~69岁",

age_group == "70-79" ~ "70~79岁",

age_group == "80+" ~ "≥80岁"

) %>% factor(levels = c("60~69岁", "70~79岁", "≥80岁")),

# GOLDGrading is an ordered factor.

GOLD = factor(GOLD, levels = c("A组", "B组", "E组")),

# Simplified variable for hospital visits frequency

hospitalization_simple = case_when(

hospitalization == "0次" ~ "0次",

hospitalization == "1次" ~ "1次",

TRUE ~ "≥2次"

) %>% factor(levels = c("0次", "1次", "≥2次")),

# Convert to binary variable(0/1)

chronic_pain_bin = ifelse(chronic_pain == "有", 1, 0),

anxiety_bin = ifelse(anxiety == "有", 1, 0)

)

# Construct a multiple linear regression model

model <- lm(adherence ~

TSK_continuous +

relevel(age_group2, ref = "60~69岁") +

relevel(hospitalization_simple, ref = "0次") +

relevel(GOLD, ref = "A组") +

chronic_pain_bin +

anxiety_bin,

data = data)

# As a result lead

write.xlsx(list("Table4" = final_table), "Table 4 - Multiple Linear Regression Analysis of Exercise Compliance.xlsx")

# Analysis of the Threshold Effect of Exercise Fear on Exercise Compliance in Elderly Patients with COPD

# Creation Time: 2025-07-21

# Load the required packages

library(tidyverse)

library(openxlsx)

library(segmented)

library(lmtest)

# Read Data

data <- read.xlsx("COPD_运动依从性.xlsx") %>% as_tibble()

# Model specification

model_linear <- lm(adherence ~ TSK_continuous, data = data)

model_segmented <- segmented(model_linear, seg.Z = ~ TSK_continuous,

psi = 20, control = seg.control(display = FALSE))

# Model Compare

lrtest <- lrtest(model_linear, model_segmented)

# Export results

write.xlsx(table5, "表5_运动依从性与运动恐惧阈值效应分析.xlsx")
